# Toward accurate molecular identification of species in complex environmental samples: testing the performance of sequence filtering and clustering methods

**DOI:** 10.1002/ece3.1497

**Published:** 2015-05-13

**Authors:** Jullien M Flynn, Emily A Brown, Frédéric J J Chain, Hugh J MacIsaac, Melania E Cristescu

**Affiliations:** 1 Department of Biology, McGill University1205 Docteur Penfield, Stewart Biology Building, Montreal, Quebec, Canada, H3A 1B1; 2 Great Lakes Institute for Environmental Research, University of WindsorWindsor, Ontario, Canada

**Keywords:** 18S rRNA, biodiversity, eDNA, high-throughput sequencing, metabarcoding, OTU

## Abstract

Metabarcoding has the potential to become a rapid, sensitive, and effective approach for identifying species in complex environmental samples. Accurate molecular identification of species depends on the ability to generate operational taxonomic units (OTUs) that correspond to biological species. Due to the sometimes enormous estimates of biodiversity using this method, there is a great need to test the efficacy of data analysis methods used to derive OTUs. Here, we evaluate the performance of various methods for clustering length variable 18S amplicons from complex samples into OTUs using a mock community and a natural community of zooplankton species. We compare analytic procedures consisting of a combination of (1) stringent and relaxed data filtering, (2) singleton sequences included and removed, (3) three commonly used clustering algorithms (mothur, UCLUST, and UPARSE), and (4) three methods of treating alignment gaps when calculating sequence divergence. Depending on the combination of methods used, the number of OTUs varied by nearly two orders of magnitude for the mock community (60–5068 OTUs) and three orders of magnitude for the natural community (22–22191 OTUs). The use of relaxed filtering and the inclusion of singletons greatly inflated OTU numbers without increasing the ability to recover species. Our results also suggest that the method used to treat gaps when calculating sequence divergence can have a great impact on the number of OTUs. Our findings are particularly relevant to studies that cover taxonomically diverse species and employ markers such as rRNA genes in which length variation is extensive.

## Introduction

**Figure 1:**
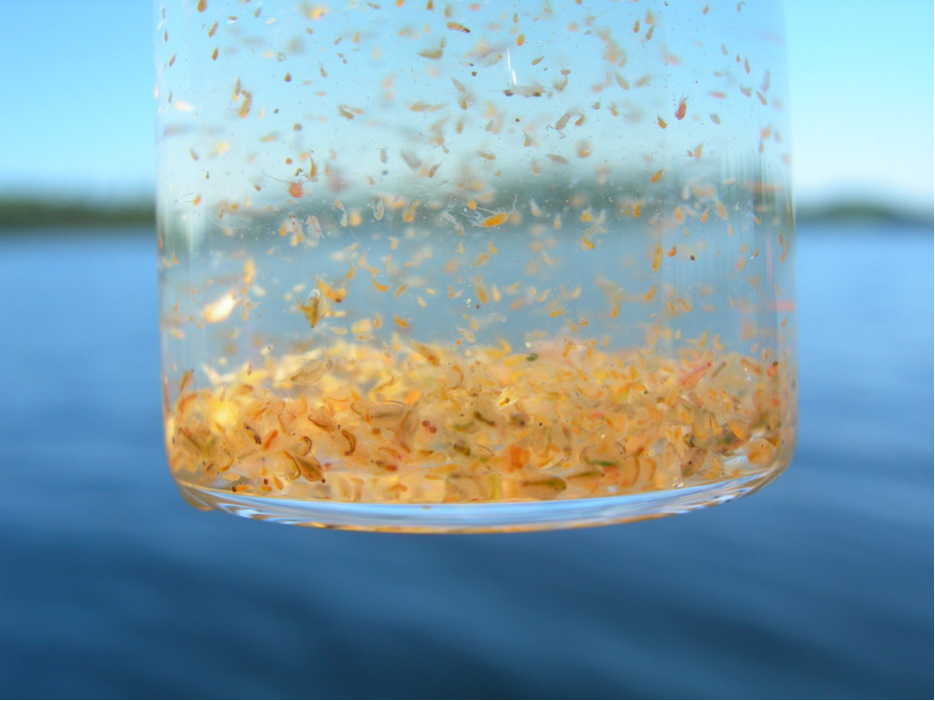
A natural zooplankton community sampled from Sudbury, Ontario, Canada.

Metabarcoding is a rapidly growing approach that provides promising opportunities to explore biological diversity in great depth. The technique combines taxonomic identification via DNA barcoding (Hebert et al. 2003) with the application of high-throughput sequencing technology to identify multiple taxa in complex biological assemblages. Identifying the community composition of an environmental sample (e.g., Fig. 1) or eDNA forms the basis of understanding for many ecological processes and ecosystem management regimes (e.g., Fonseca et al. 2010; Pawlowski et al. 2014), with applications including diet assessment and community response to toxic conditions (e.g., Pompanon et al. 2012; Chariton et al. 2014). However, data processing for a metabarcoding study can be a daunting task for ecologists who wish to identify the species present in a sample, and even for bioinformaticians trying to validate their methods (McPherson 2009). In order to estimate species diversity in a complex sample, sequences are clustered into operational taxonomic units (OTUs), which are used as a proxy for species. Diversity estimates can vary greatly depending on the methods used (Bachy et al. 2013; Egge et al. 2013), and therefore, robust assessments of various methods are valuable to guide the selection of optimal procedures for a particular study.

Several components of sequence data processing can strongly impact the results of a metabarcoding study. Firstly, the filtering of raw sequence reads is important for the removal of sequences potentially containing errors. A second important factor is whether unique sequences that are represented by only a single read, known as singletons, should be included or removed in the analysis. The choice of the clustering algorithm that groups sequences to generate OTUs is also a very important component. Finally, a rather neglected factor is the “identity definition”, which considers how alignment gaps are treated when calculating sequence divergence. This clustering parameter is particularly important when analyzing markers that show extensive length variation and evolve with frequent insertions and deletions.

### Filtering

Several filtering algorithms have been developed to remove low quality, erroneous, or artefactual sequences such as chimeric sequences formed during PCR (e.g. RDP, Cole et al. 2009; USEARCH, Edgar 2010; SeqTrim, Falgueras et al. 2010; CANGS, Pandey et al. 2010; PyroCleaner, Mariette et al. 2011; AmpliconNoise Quince et al. 2011). Despite constant improvement of these methods, insufficient removal of such artefactual sequences in biodiversity studies has likely caused considerable inflation of some diversity estimates (Kunin et al. 2010). Several studies that applied metabarcoding have reported a much higher diversity of species than expected based on traditional sampling and morphological identification – contributing to the observation of the so-called rare biosphere consisting of many low abundance species. Further verification has shown that some of these estimates are likely not representative of legitimate biodiversity, but rather reflect artifact generated as a consequence of amplification and sequencing errors combined with inadequate data processing procedures (Kunin et al. 2010; Behnke et al. 2011; Bachy et al. 2013). However, the extent to which metabarcoding methods are prone to generating highly inflated biodiversity estimates remains largely unexplored. Another contentious issue is the removal of singletons to reduce the impact of spurious errors (Kunin et al. 2010; Behnke et al. 2011), although some authors argue that singletons may be important for the detection of rare species in a sample (Zhan et al. 2013).

### Clustering

After filtering, sequences are generally clustered into OTUs, sometimes referred to as “OTU-picking”. This step groups similar sequences to account for minor differences between reads stemming from biological variation (e.g., polymorphism in sequences from individuals of the same species, or between gene copies within an individual) and from PCR or sequencing errors. Numerous clustering programs that apply different algorithms have been developed (Table1). Most de novo clustering algorithms (without the use of reference sequences known a priori) use a hierarchical or greedy heuristic approach (Sun et al. 2012), although a few new developments use alternative statistical or modularity-based approaches (e.g., CROP, M-Pick, SWARM, Table1). In general, hierarchical algorithms compute sequence divergence between all pairs of sequences – which is very computationally demanding – producing a distance matrix before generating OTU clusters. Greedy heuristic algorithms perform fewer pairwise comparisons to estimate optimal clustering parameters, improving computational efficiency (Sun et al. 2012). In this study, we compare three commonly used algorithms representing the two major types of clustering options; hierarchical clustering algorithm mothur and greedy heuristic algorithms UCLUST and UPARSE. We also chose these because they have clear documentation available and have adjustable parameters, allowing us to test different identity definitions.

**Table 1:** List of different clustering algorithms (not exhaustive). Identity definitions: *no gaps* = gaps are not included in the identity calculation; *one gap* = a gap of any size is treated as a single mutational difference; *each gap* = each nucleotide in the gap is treated as an additional mutational difference

Algorithm name	Algorithm type	Identity definition(s) used/available
mothur (Schloss et al. 2009)	Hierarchical	Default is *one gap*; other options include *each gap* and *no gaps*
UCLUST (Edgar 2010)	Greedy heuristic	*Each gap* definition is used in most recent version. Older versions: other definitions including *one gap* and a definition similar to *no gaps*.
UPARSE (Edgar 2013)	Greedy heuristic	*Each gap* definition used; user cannot change
CD-HIT (Li and Godzik 2006)	Greedy heuristic	Gaps penalized only in longer sequence of pairwise comparison; user cannot change
ESPRIT (Sun et al. 2009)	Hierarchical	*One gap*; user cannot change
ESPRIT-Tree (Cai and Sun 2011)	Hierarchical but pairwise comparisons are not exhaustive	*Each ga*p; user cannot change
CROP (Hao et al. 2011)	Bayesian approach	*One gap*; user cannot change
TSC (Jiang et al. 2012)	Step 1: hierarchical 2: greedy heuristic	Directly from alignment algorithm; user cannot change
M-pick (Wang et al. 2013)	Modularity based	*One gap*; user cannot change
MSClust (Chen et al. 2013b)	Greedy heuristic	Directly from alignment algorithm; user cannot change
SWARM (Mahé et al. 2014)	Agglomerative	*One gap*

Different clustering methods can lead to extensively different biodiversity estimates (Bachy et al. 2013). These methods vary in user-friendliness, accuracy, computational speed, and memory usage, and their suitability for a particular study can depend on the target taxa, markers, type of samples, sequencing methods, and goals of analyses. This makes choosing an appropriate clustering method challenging, especially in the absence of comprehensive performance tests and robust biodiversity censuses of the given samples.

### Identity definitions

An important factor to consider when clustering sequences is the identity definition, which is used in the calculation of divergence between sequences during OTU assignment. This parameter is especially important for clustering nonprotein coding markers such as variable regions of ribosomal RNA genes that evolve with frequent insertions and deletions (indels) (Wuyts et al. 2000; Englisch et al. 2003) and whose length can vary between taxa by hundreds of nucleotides (Crease and Taylor 1998; Choe et al. 1999). In addition to the presence of a wide spectrum of evolutionary informative gaps, artificial gaps can be introduced by homopolymer misreads, a common type of error in sequencing data, specifically with pyrosequencing (Huse et al. 2007). Although this artifact may be less prevalent with other high-throughput sequencing platforms, pyrosequencing remains highly used for the generation of long reads that span variable regions of the 18S gene. Markers that exhibit significant length variation have specific computational requirements. Indels cause gaps in the sequence alignment, and how these gaps are scored greatly affects the calculated divergence between sequences. The computational aspects related to handling gaps have largely been overlooked by the metabarcoding community, despite the common use of ribosomal markers in metabarcoding studies (Fonseca et al. 2010; Pawlowski et al. 2014). The effect of using different identity definitions on diversity estimates has only been investigated on prokaryotic 16S sequences (Schloss 2010). This study found that length variation in the markers had an impact on sequence divergence calculations, but the effect of different gap treatments did not greatly impact diversity estimates (Schloss 2010). However, this potential problem has not been evaluated on complex eukaryotic communities or on markers with extensive length variation such as 18S. Thorough investigations on the effect of gap treatment on biodiversity estimates are largely precluded by technical limitations. It is typically not obvious how different clustering algorithms treat gaps or missing data. Most importantly, different algorithms have different default settings for the treatment of gaps, which may or may not be changeable by the user, making direct comparisons of algorithms challenging (Table1).

There are typically a few identity definitions that can be implemented in clustering algorithms. Gaps can be excluded from the calculation altogether, a gap of any length can be treated as a single mutational difference, or each nucleotide in a gap can be treated as a separate mutational difference (Schloss 2010). The treatment of gaps should reflect the molecular evolution of the marker as the objective is to distinguish species based on sequence differences. Gap treatment is therefore very important when clustering sequences that contain many or large indels.

### Comparing workflows

The few studies that compare workflows – defined here as the combination of data processing procedures that result in OTUs – and specifically the use of different clustering methods (Table2) provide conflicting results that can leave researchers overwhelmed with their decision on how to process metabarcoding data. Most verification tests have been carried out with prokaryotic sequences from mock datasets (simulated sequences or sequences from a database), mock communities (sequenced DNA from known species), or natural communities (for which the ground truth composition is difficult to estimate) (Bachy et al. 2013; Chen et al. 2013a; Wang et al. 2013). The OTU number generated has often been used as a proxy for the accuracy of the workflow and the workflows that produce the least overestimation of diversity are assumed to be the best. Other studies have compared the quality of OTUs produced from different workflows, but have not related OTUs to taxonomy (Edgar 2013; Table2). This can be problematic even if an accurate ground truth is known because it is possible that multiple OTUs will be generated for some taxa simply due to biological variation, while other taxa are completely missed (e.g., not amplified or removed during data processing). Although clustering methods have been compared on a eukaryotic community with known diversity (Bachy et al. 2013; Table2), the community examined had limited taxonomic breadth (a single order), and not all parameters were explored. As environmental samples can be composed of highly divergent taxa, the efficacy of clustering methods is better assessed using a diverse mock community consisting of a wide range of taxonomic groups. In this way, the most suitable workflow to reduce both oversplitting (i.e., producing multiple OTUs representing the same species) and undersplitting OTUs (i.e., closely related species being placed in the same OTU because of insufficient taxonomic resolution) can be evaluated.

**Table 2:** Previous studies that have compared clustering methods (not exhaustive)

Reference	Relevant methods compared	Marker(s) and data used	Performance measure(s)	Conclusions
Barriuso et al. (2011)	mothur, ESPRIT, CROP, UCLUST, RDP clustering	16S sequences	OTU number compared to expected	RDP, ESPRIT, UCLUST produced acceptable results, CROP produced anomalous results
		Synthetic and natural community data		mothur unable to process large datasets
Sun et al. (2012)	MSA vs. PSA^1^; hierarchical vs. greedy heuristic clustering	16S sequences	OTU number	Although PSA does not consider secondary structure like MSA can, PSA still produced more reliable estimates with 16S sequences
	CD-HIT, UCLUST, ESPRIT, MUSCLE, ESPRIT-Tree	Simulation and natural community data	NMI^2^ and F-score^2^	Hierarchical clustering algorithms performed better
Edgar (2013)	UPARSE, AmpliconNoise^3^, mothur, QIIME^4^ (implementing UCLUST)	16S sequences	OTU number	UPARSE performed best: most perfect and good sequences and fewest chimeric sequences
		Two mock communities and natural community data	Classified OTUs as perfect, good, noisy, chimeric	UPARSE OTUs approached 1:1 correspondence with species in mock community
Chen et al. (2013b)	ESPRIT, ESPRIT-Tree, mothur, muscle+mothur, CROP, CD-HIT, UCLUST, SLP^5^, DNAClust^6^, GramCluster^7^	Dataset of 16S sequences of known microbial species	NID^5^ score	With default parameters, the methods tended to inaccurately estimate number of OTUs
		Simulated 16S datasets	OTU number compared to expected	
Bachy et al. (2013)	MSA+mothur, AmpliconNoise, USEARCH workflow, CD-HIT-OTU	18S and ITS sequences from a mock community of protist morphotypes	OTU number compared to expected from morphology and map to reference dataset	Great differences in OTU number, some methods overestimating by an order of magnitude
				Denoising methods tended to underestimate some of the species richness.
Yang et al. (2013)	USEARCH+CROP, Denoiser+UCLUST, OCTUPUS^8^	18S and CO1 sequences	OTU number	Pipelines produced similar results for community composition
		Natural community data		OCTUPUS appeared to inflate diversity
Bonder et al. (2012)	Filtering: none, chimera removal, denoising, denoising + chimera removal	16S sequences	OTU number compared to expected	CD-HIT, UCLUST, ESPRIT-Tree performed well
	Clustering: UCLUST, mothur, ESPRIT-Tree, CD-HIT, QIIME	Mock community and natural community datasets	NMI score	Filtering required for accurate OTU estimates
May et al. (2014)	Filtering: none, chimera removal, denoising, denoising then chimera removal, chimera removal then denoising	16S sequences	OTU number compared to expected	The choice and order of filtering options have a great impact on clustering results
	Clustering: 11 different clustering algorithms were evaluated	Mock community datasets and simulated datasets	NMI score	After chimera removal and denoising, the performance of the different clustering algorithms was similar

1 MSA – multiple sequence alignment; PSA – pairwise sequence alignment (when comparing sequences during clustering).

2 Metric of cluster quality and proper assignment of sequences; generally requires a ground truth composition to determine.

3 Algorithm that denoises reads before further processing (Quince et al. 2011).

4 Pipeline that implements a variety of tools for data processing (Caporaso et al. 2010).

5 Single linkage preclustering; a method that attempts to reduce noise to minimize OTU estimate inflation (Huse et al. 2010).

6 Greedy heuristic algorithm (Ghodsi et al. 2011).

7 Greedy heuristic algorithm based on a grammar distance metric (Russell et al. 2010).

8 Fonseca et al. (2010).

In the present study, we focus on metabarcoding zooplankton using the hypervariable V4 region of the 18S rRNA gene, a region prone to expansion and contraction via slippage mutations and characterized by great length variation across eukaryotic taxa (Hancock 1995; Hwang et al. 2000). For example, between the families Artemiidae and Daphniidae (both branchiopod crustaceans), the length of the V4 region differs by up to 237 nucleotides (Crease and Taylor 1998). We use a mock community with morphologically identified zooplankton species and perform downstream taxonomic classification of OTUs to assess the accuracy of different workflows in estimating biodiversity. Furthermore, we use a natural zooplankton community to explore the range of OTU numbers produced by the various workflows. We evaluate workflows consisting of stringent and relaxed filtering, each with singletons included and removed – producing four datasets for both the mock and natural community. We then cluster each of these datasets using mothur, UCLUST, and UPARSE algorithms. At the clustering stage we also test three different identity definitions in order to evaluate the effect of gap treatment on the overall efficacy of species identification.

## Materials and Methods

### Mock community assembly

The mock community included 61 zooplankton species from broad taxonomic groups encompassing three eukaryotic phyla: Arthropoda (subphylum Crustacea, 51 species), Chordata (subphylum Tunicata, two species), and Mollusca (eight species). The crustaceans, making up a majority of the community belonged to six major groups: Amphipoda, Anostraca, Cirripedia, Cladocera, Copepoda (calanoids, cyclopoids, harpacticoids), and Decapoda, (Table S1). Each species in the community was represented by a single individual, which was dissected to roughly equal volume corresponding to a medium size cladoceran. All individuals included were identified either to species or genus level by taxonomists, with eight exceptions that were identified to family level (e.g., decapod larvae, Table S1). We ensured that these specimens were genetically diverged from other community members so that they could be unambiguously identified (Table S2). All individuals were washed with distilled water prior to inclusion in the community. Due to the relatively large number of individuals involved, the community was assembled in four separate microcentrifuge tubes, each containing approximately 15 individuals. Following assembly, any fluid remaining from the washing process was removed by centrifugation at 6797 g for 3 min and extraction of the supernatant was performed with a fine pipette. The supernatant was subsequently examined under the microscope to ensure that no tissue or animals were removed during the concentration process.

### DNA extraction, PCR amplification, and pyrosequencing

Total genomic DNA was isolated independently from the tissue in the four tubes using DNeasy Blood and Tissue Kits (Qiagen, Venlo, Limburg, Netherlands) following the manufacturer's protocol. The primer pair developed by Zhan et al. (2013) (Uni18S: AGGGCAAKYCTGGTGCCAGC; Uni18SR: GRCGGTATCTRATCGYCTT) was used to amplify approximately 400–600 bp of the hypervariable V4 region of the 18S rRNA gene. Preliminary testing with single species extraction and amplification confirmed that all of the taxonomic groups included in the community could be amplified by this primer set. The 454 FLX adapters (adapter A: CCATCTCATCCCTGCGTGTCTCCGACTCAG, adaptor B: CCTATCCCCTGTGTGCCTTGGCAGTCTCAG) were added to the 5′ end of the forward and reverse primers, respectively, to make them compatible with pyrosequencing procedures. Eight replicate PCR mixtures (25 *μ*L each) were prepared for each of the four independent extractions in an attempt to reduce the effect of PCR biases that may have occurred in any given reaction. Each reaction consisted of approximately 100 ng of genomic DNA, 1× PCR buffer, 2 mmol/L of Mg^2+^, 0.2 mmol/L of dNTPs, 0.4 *μ*mol/L of each primer, and 2 units of *Taq* polymerase (Genscript, Piscataway, NJ, USA). PCR cycling parameters consisted of an initial denaturation step at 95°C for 5 min, followed by 25 amplification cycles of 95°C for 30 sec, 50°C for 30 sec, 72°C for 90 sec, and a final elongation step at 72°C for 10 min. All PCR products were cleaned to remove short products using Solid Phase Reversible Immobilisation (SPRI) paramagnetic bead-based method (ChargeSwitch, Invitrogen, Carlsbad, CA, USA). The quality and quantity of DNA was assessed using gel electrophoresis and Quant-iT PicoGreen dsDNA Assay kit (Invitrogen). All cleaned PCR products (32 total) were then pooled together in equimolar concentrations before pyrosequencing at ½ PicoTiter plate scale. Pyrosequencing was performed using 454 FLX Adapter A on a GS-FLX Titanium platform (454 Life Sciences, Branford, CT, USA) by Genome Quebec. Pyrosequencing remains the most accessible technology able to sequence the read lengths necessary to provide species diagnosis with this marker. Data were deposited in the Sequence Read Archive (SRA, http://www.ncbi.nlm.nih.gov/sra) under Accession Number SRX884895.

### Natural community

We also applied our workflows on natural community sequence data (SRA Accession Number SRX889243) generated by Zhan et al. (2013) from a zooplankton sample collected from Hamilton Harbour, Ontario, Canada. Procedures prior to sequencing (DNA extractions, PCRs, etc.) were similar to those described for the mock community. Moreover, all analytical procedures were the same as those used for the mock community.

### Data filtering

In order to assess the outcome of including more reads at the cost of potentially retaining more artifacts, we filtered raw sequence data using either a stringent or relaxed procedure (Table3). The stringent procedure was implemented in USEARCH (Edgar 2013). The relaxed procedure was implemented through RDP pyrosequencing pipeline (https://pyro.cme.msu.edu/index.jsp), a user-friendly platform, applying the filtering method used by Zhan et al. (2013). An important difference between the stringent and relaxed filtering procedures is the way in which sequences were trimmed – our stringent filtering procedure trimmed all sequences to 400 bp (sequence quality dropped beyond this length) and removed sequences of length <400 bp, while our relaxed filtering procedure retained reads of variable length (ranging from 250 to 600 bp). The type of quality filtering also differed – our stringent method used the maximum expected error as a threshold for removing low-quality sequences, whereas our relaxed procedure used average quality scores as a filtering criterion. It has been argued that using average quality scores results in a higher chance of retaining sequences with true errors (Edgar 2013).

**Table 3:** Main characteristics of stringent and relaxed filtering procedures

Stringent filtering (USEARCH)	Relaxed filtering (RDP)
Primer mismatches removed	Primer mismatches removed
Sequences <400 bp removed and remaining sequences trimmed to 400 bp	Sequences <250 bp or >600 bp removed
Sequences containing ambiguous nucleotides (Ns) removed	Sequences containing ambiguous nucleotides (Ns) removed
Sequences with expected error >0.5 removed	Sequences with average quality <20 removed
Chimeras removed with UCHIME^1^	Chimeras removed with UCHIME^1^

1 Except for datasets clustered with UPARSE.

Denoising is another quality control method that clusters the raw flowgrams that give intensities (homopolymer length) of the reads, before converting to nucleotide sequences in an attempt to reduce homopolymer read errors (Quince et al. 2009). However, with large datasets, denoising requires extensive computational memory and is not always feasible except with large computer clusters. Therefore, we decided to test the two filtering methods described above, which are practical for most independent researchers.

**Figure 2:**
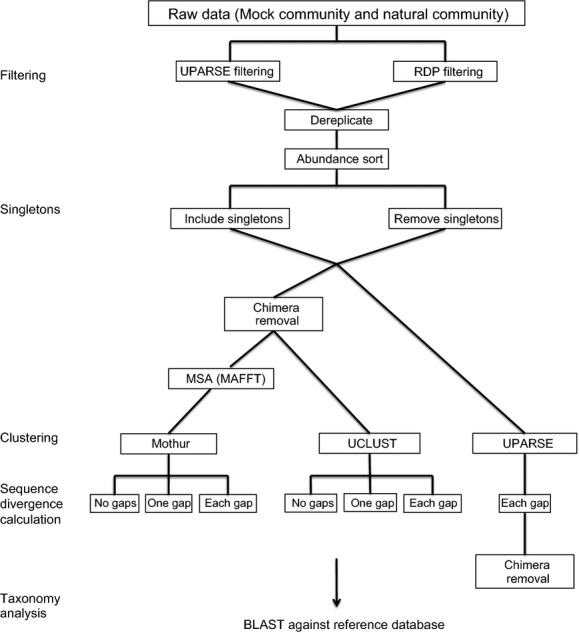
Data analysis methods. MSA refers to multiple sequence alignment.

After initial filtering in USEARCH or RDP, identical reads were dereplicated using USEARCH (Fig.2), a process in which identical reads are collapsed to a single read for more efficient clustering. In order to test if singletons can provide relevant information or whether they only add noise, datasets were analyzed both with and without singletons. Therefore, four sequence datasets for each the mock community and the natural community were generated for subsequent analysis: one filtered by RDP and one filtered by USEARCH, each with and without singletons. Chimeras were removed using UCHIME (Edgar et al. 2011) for all datasets before clustering, except for the datasets clustered with UPARSE, where chimera removal occurs simultaneously with OTU-picking and a final chimera check with UCHIME is performed after clustering (Fig.2).

### Clustering

We chose to test the performance of three commonly used clustering algorithms: mothur, which performs hierarchical clustering, as well as UCLUST and UPARSE, which perform greedy heuristic clustering. Mothur takes as input a multiple sequence alignment and generates clusters after building a distance matrix of all pairwise comparisons of sequences (Schloss et al. 2009). We performed multiple sequence alignments with default settings in MAFFT v7.150b (Katoh and Standley 2013) before inputting the alignments into mothur, as per Bachy et al. (2013). UCLUST takes as input sequences in order of decreasing abundance, with the assumption that more abundant sequences are more likely to represent genuine sequences as opposed to artifacts (Sun et al. 2012; Edgar 2013). The most abundant sequence becomes the founder of the first cluster, and each subsequent sequence is compared in a pairwise manner, either joining an existing cluster or becoming the founder of a new cluster if it is not similar enough to the founder sequence of the existing clusters. UPARSE functions in a similar way as UCLUST except that a maximum parsimony score is calculated when comparing pairs of sequences (Edgar 2013). This score is used both to determine whether or not the sequence should join the query cluster and to determine whether it is chimeric.

### Identity definitions

Because mothur and earlier versions of UCLUST allow the user to select the identity definition to calculate sequence divergence, we used these two algorithms to test the effects of different identity definitions on the results of OTU-picking. We adopt the terminology used for mothur and refer to the definitions as *no gaps* when gaps in the alignment are excluded from the calculation, *one gap* when a gap of any length is treated as a single mutational difference, and *each gap* when each nucleotide in a gap is treated as a separate mutational difference. In UCLUST, we used the CD-HIT definition for *no gaps*, MBL for *one gap*, and All-diffs for *each gap*. USEARCH v.5.2 was used for the implementation of UCLUST because this version allows the user to change the identity definition and more recent versions do not. UPARSE does not allow the user to change the identity definition, so only the default of *each gap* was used. All datasets were clustered at a 3% divergence threshold with each of the three clustering algorithms. The RDP-filtered datasets including singletons were not clustered with mothur because these datasets retained a large number of sequences (169,807 for the mock community and 130,433 for the natural community), and it was not possible to compute a matrix of pairwise differences due to computational memory requirements. We initially evaluated OTU numbers for each workflow using 3%, 4%, and 5% divergence thresholds, but only report results for 3%. Our preliminary test indicated that OTU number differences between workflows were greater than that between divergence thresholds. Testing the appropriateness of different divergence thresholds for clustering a complex zooplankton community is thoroughly addressed in the companion paper Brown et al. using a series of complex mock communities that include various levels of genetic variation (interspecific, intrapopulation, and intra-individual).

### Taxonomic classification

In order to compare methods in a consistent fashion, OTUs from the mock community datasets were classified using a reference BLAST database (Altschul et al. 1990) of 18S sequences, which was constructed with sequences from the nucleotide database from NCBI and the SILVA database (Quast et al. 2013). For the mock community species that were not in one of these databases, the most closely related species (some of which were only in the same family) was designated as a reference sequence if it was diverged from the other reference sequences in the community (beyond the divergence threshold used). Therefore, all 61 species had a reference sequence included in our database. All downloaded reference sequences were aligned (MAFFT v7.150b) and trimmed around the V4 region to produce our reference BLAST database against which we compared OTUs. We identified the species from our community that were putatively successfully amplified by performing BLAST searches using all unfiltered reads against our reference database. We were able to unambiguously recover 46 community specimens, whereas 15 were absent from our data. We removed from the analysis three species that could not be distinguished by our 3% divergence threshold (Table S3), leaving us with 43 reference sequences. This ensured that our analysis only took into account those species from the community that were actually amplified. After each workflow was performed, the representative OTU sequences were taxonomically classified based on their best BLAST hit against the reference database. A positive identification consisted of a BLAST hit with at least 90% identity and an alignment length of at least 200 nucleotides with a reference database sequence. These parameters were relatively relaxed to accommodate congeneric or confamilial reference sequences, but were checked to ensure the identity matched the expectation based on the relatedness of the corresponding reference sequence (Table S2).

To compare the accuracy in estimating biodiversity in the mock community, the proportion of species recovered was assessed for each workflow, which was used to calculate a “precision” score. Precision was calculated as the number of species recovered from the reference database divided by the total number of OTUs generated. A precision score of 1.0 would signify that all OTUs correctly corresponded to the species included in the mock community, with no extra OTUs. A low precision would signify the presence of many spurious OTUs and could result from artefactual sequences (producing nontarget OTUs), and/or from multiple OTUs being generated for the same species (oversplitting). For example, if a workflow recovered 40 species from the database but produced 70 OTUs, the precision would be 40/70 (0.57). To rigorously compare the three identity definitions, precision was also calculated for each of the 10 higher level taxonomic groups included in the mock community using datasets clustered by mothur.

## Results and Discussion

### Stringent or relaxed filtering?

OTU numbers varied by orders of magnitude depending on the combination of data filtering and clustering methods used (Table4). For the mock community, the most stringent workflow (USEARCH filtering, singletons removed, UPARSE clustering) recovered 60 OTUs whereas the most relaxed combination (RDP filtering, singletons included, UCLUST clustering with *each gap* identity definition) recovered 5068 OTUs. When singletons were removed, however, stringent and relaxed filtering workflows produced more comparable results ranging from 60 to 263 OTUs (Table4). The largest differences came from the RDP-filtered datasets that were clustered with mothur and UCLUST using the *each gap* identity definition. This workflow recovered the highest OTU numbers (262 and 263 OTUs, respectively) due to the combination of RDP filtering, which does not trim sequences to a uniform length, and the *each gap* definition, in which each nucleotide in a gap contributes to sequence divergence during clustering.

**Table 4:** Mock and natural community OTU results. The number of preprocessed reads indicates the reads before quality and length filtering and the number of processed sequences is the number of sequences after filtering and dereplication. First number represents the total number of OTUs; the number in brackets represents OTUs that matched the target species in the mock community; in bold are numbers of species detected from the mock community

	USEARCH filtering						RDP filtering				
	Singletons removed			Singletons included			Singletons removed			Singletons included	
Mock community (61 species) 427,868 preprocessed reads											
Processed sequences	6490			19,881			22,923			169,807	
ID def	mothur	uclust	uparse	mothur	uclust	uparse	mothur	uclust	uparse	uclust	uparse
No gaps	63 (57) **40**	64 (58) **40**	–	83 (68) **42**	101 (86) **42**	-	70 (60) **40**	78 (68) **41**	–	716	–
One gap	70 (64) **40**	62 (56) **40**	–	98 (83) **42**	94 (79) **42**	-	75 (65) **40**	79 (68) **40**	–	1025	–
Each gap	79 (73) **40**	68 (62) **40**	60 (54) **40**	137 (121) **42**	114 (99) **42**	84(69) **42**	262 (239) **41**	263 (241) **41**	75 (62) **41**	5068	647
Natural community 497,806 preprocessed reads											
Processed sequences	2731			15,806			7080			130,433	
ID def	mothur	uclust	uparse	mothur	uclust	uparse	mothur	uclust	uparse	uclust	uparse
No gaps	22	22	–	96	147	–	28	42	–	2723	–
One gap	33	33	–	227	160	–	46	48	–	5471	–
Each gap	43	44	24	312	243	63	247	230	38	22,191	1174

**Figure 3:**
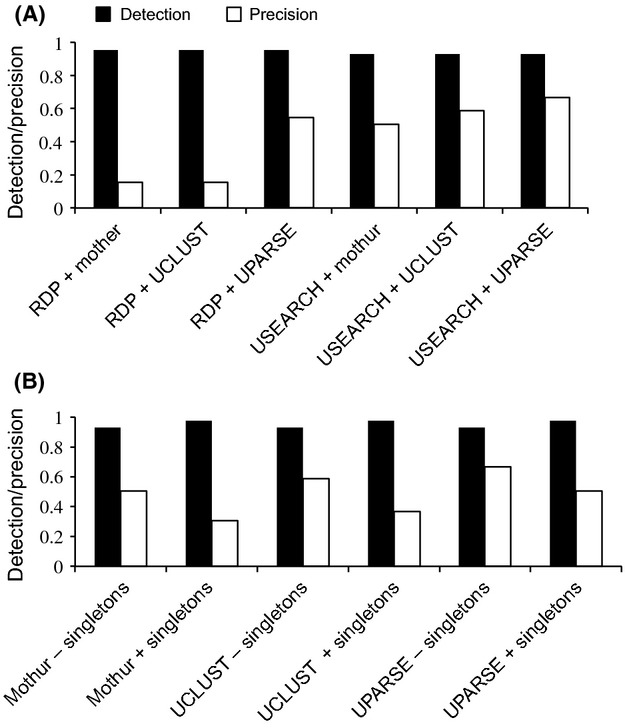
Species detection and precision across workflows. Species detection is the ratio of the number of species recovered and the number of species in the mock community database, whereas precision is the ratio of the number of species recovered and the number of OTUs. (A) The combination of relaxed (RDP) and stringent (USEARCH) filtering methods with clustering algorithms. Results shown for the mock community dataset with singletons removed, and *each gap* identity definition was used for all clustering algorithms. (B) The combination of removing singletons (− singletons) and including singletons (+ singletons) with all clustering algorithms. Results shown for the mock community dataset filtered with USEARCH and clustering with *each gap* identity definition.

**Figure 4:**
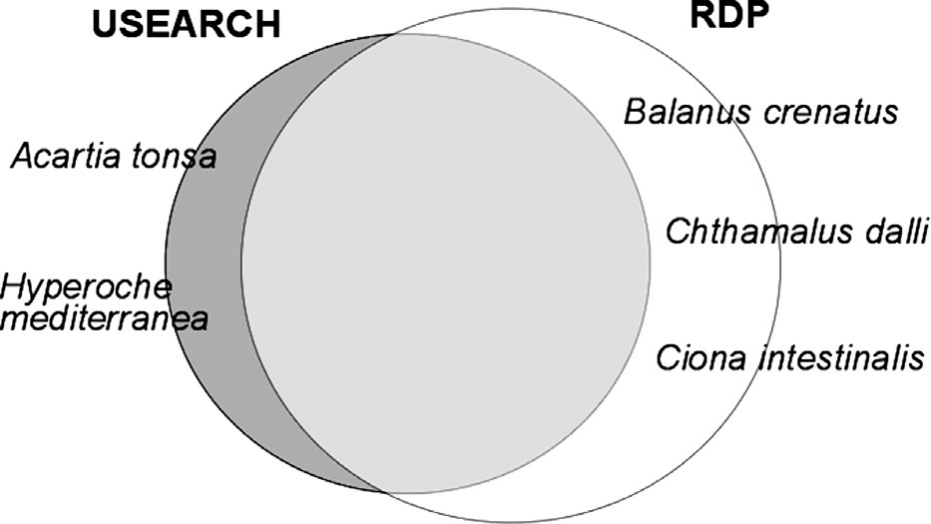
Species detected unique to the particular filtering method. Stringent (USEARCH) versus relaxed (RDP) filtering – both with singletons removed and clustered with UPARSE. The size of the circle corresponds with the number of species recovered.

Despite the variation in OTU numbers retrieved from the various workflows, the actual number of target species recovered did not differ greatly, ranging between 40 and 42 of a possible 43 (Table4). None of the workflows tested recovered all 43 species, but all 43 species were recovered by at least one workflow. However, stringent filtering consistently had higher precision (Fig.3A) whereas relaxed filtering repeatedly formed multiple OTU clusters matching the same species – suggesting oversplitting of clusters – as well as more OTUs that did not match species from the mock community. Generating more OTUs that represent the same species reflects increased sequence variation either produced by genuine biological variation or sequencing artifacts. Most of the OTUs that did not match reference sequences represent artifacts with no BLAST hits, contaminants that match other zooplankton species, or ambiguous “uncultured eukaryote” sequences. Contaminant species (e.g., an annelid) that were not targeted by our primers were more often detected with relaxed filtering compared to stringent. We found differences in the recovery of five mock community species when comparing relaxed and stringent filtering (Fig.4), with relaxed filtering having only a slightly higher proportion of species recovered (Fig.3A). Stringent filtering consistently produced OTU numbers closer to the number of species in the community than relaxed filtering. Additionally, OTU number was less impacted (inflated) by the inclusion of singletons with stringent filtering (Table4). Therefore, we highly recommend the use of stringent filtering when metabarcoding approaches are used to generate accurate biodiversity estimates. We found that stringent filtering reduces redundancy and noise and reduces the problem of generating inflated numbers of OTUs, without considerably decreasing the number of species that could be recovered.

### Include or remove singletons?

In the workflows that included singletons, relaxed filtering had a much greater number of singleton reads (108,663) compared to stringent filtering (13,241). Including singletons with relaxed filtering also resulted in very high OTU numbers (Table4). However, including singletons in the mock community generally did not increase the proportion of species detected (Fig.3B). Instead, we found that including singletons mainly increased the number of OTUs representing the same species that were already detected when singletons were not included, decreasing precision. This suggests that most singletons are either rare alleles or the products of artifacts or sequencing errors, in agreement with past findings (Tedersoo et al. 2010). In other words, low-quality sequences that contained errors but originated from the same species (same individual) were clustered into different OTUs because they contained sufficient differences beyond the divergence threshold. Datasets with singletons also generated more OTUs that did not match a target species (e.g., 15 vs. six for the USEARCH filtering and UPARSE clustering workflows).

Including singletons did allow for the recovery of two species (*Ciona intestinalis* and *Chthamalus dalli*) that were not recovered when singletons were removed after USEARCH filtering. These species were only represented by a single read, probably due to inefficient amplification despite doing multiple independent PCRs. In general, however, the retention of singletons had a higher impact on decreasing precision (more OTUs) than it did on increasing species detection. Depending on the type of study and the research goal, the trade-off between generating accurate OTU numbers and retaining the ability to detect genuine rare species needs to be evaluated. It is important to keep in mind that our mock community had only one individual per species and included approximately the same volume of tissue for each individual. Singletons therefore may be more important in a situation where some species are present at a much lower abundance than others. With the increased read depth of other platforms (e.g., Illumina), singletons are even more likely to be artifacts and may be more readily discarded for biodiversity assays (Edgar 2013).

### Clustering algorithms

We tested the hierarchical clustering algorithm mothur and greedy heuristic algorithms UCLUST and UPARSE. In general, mothur produced results comparable to UCLUST both in terms of OTU number and precision within the same workflow (Table4, Fig.3). Hierarchical clustering with mothur requires a multiple sequence alignment and pairwise distance matrix calculation before clustering, which takes much more time and computational resources than the greedy heuristic algorithms UCLUST and UPARSE. Clustering with mothur took hours for most datasets, compared to seconds for UCLUST and minutes for UPARSE. Previous work has shown that hierarchical clustering produced better results for bacterial 16S sequences (Sun et al. 2012), but our study shows greedy heuristic clustering to be comparable when both methods start from the same set of filtered 18S sequences. Therefore, greedy heuristic clustering may be sufficiently accurate for a eukaryotic metabarcoding study. However, using a multiple sequence alignment with a reference database and with a model that takes secondary structure of the rRNA molecule into consideration may produce more accurate results with mothur (Schloss 2010). These steps are widely practiced for bacterial 16S sequences and may be possible for eukaryotic groups with sequence information, but this would still not overcome the drawback of relying on a high-quality multiple sequence alignment of sequences of a highly polymorphic marker to calculate sequence divergence (Goeker et al. 2010).

There were no differences in species detection between the three clustering algorithms in datasets generated with stringent filtering, with or without singletons. UPARSE gave the highest precision and OTU number closest to species number than the other clustering algorithms, even in workflows with relaxed filtering and including singletons (Fig.3). For these reasons, we recommend the use of UPARSE for clustering.

### Identity definitions

**Figure 5:**
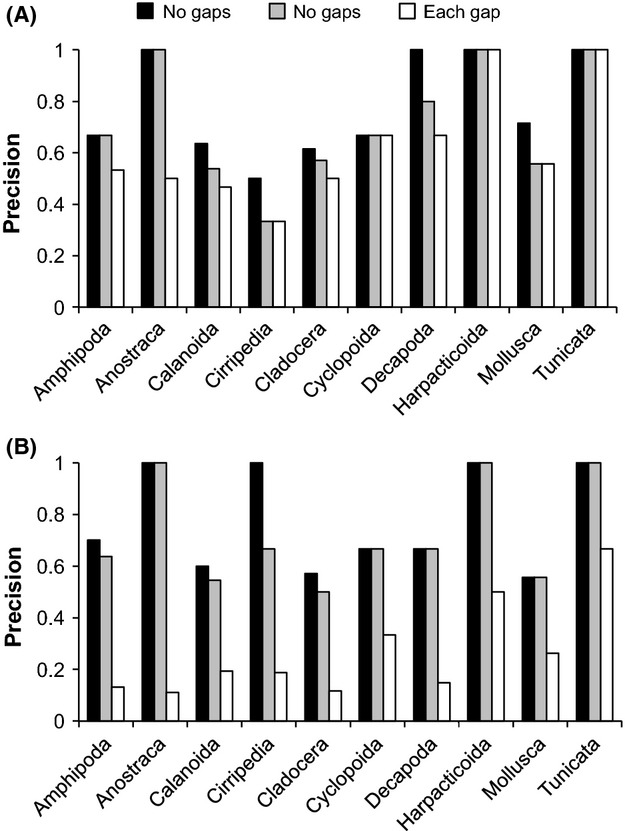
Precision comparisons of methods of calculating sequence divergence. Precision is the ratio of the number of species recovered and the number of OTUs. (A) USEARCH filtered data with singletons removed and clustered by mothur with all identity definitions. (B) RDP filtered data with singletons removed and clustered by mothur with all identity definitions.

We tested three methods for calculating sequence divergence during clustering; *no gaps*, *one gap*, and *each gap* identity definitions. The *each gap* definition tended to produce the most OTUs, *no gaps* the least, and *one gap* intermediate (Table4). *Each gap* produced much higher OTU numbers than the other definitions especially under relaxed filtering (e.g., 262 for mothur with *each gap* vs. 70 and 75 for *no gaps* and *one gap*, respectively). Precision also followed a similar pattern: highest for *no gaps,* lowest for *each gap,* and intermediate for *one gap*, with differences most pronounced with relaxed filtering (Fig.5). There were greater differences between definitions when singletons were included (Table4), likely because singletons are more likely to represent erroneous sequences (Edgar 2013) that could contain artificial indels.

The *each gap* definition produced inflated OTU numbers because terminal gaps are included in the calculation of sequence divergence with the algorithms we used. Terminal gaps are created in an alignment after stringent filtering (all sequences trimmed to 400 bp) when sequences contain internal indels, and also for relaxed filtering when sequences are different lengths. This often resulted in the formation of separate OTUs for sequences that are otherwise similar but differ in aligned length. This is the reason why the UPARSE manual clearly urges the user to input globally alignable sequences without terminal gaps. However, with the large quantity of data produced in metabarcoding studies, it is generally not practical to accurately align all sequence reads before trimming. In comparing each of the identity definitions when sequences were clustered with UCLUST after RDP filtering, *each gap* produced more OTUs that differed mainly in length. Therefore, *no gaps* and *one gap* allow similar sequences that differ primarily in length to be clustered together, whereas *each gap* produces multiple clusters containing similar sequences that differ only in length. Differences between identity definitions were more subtle with stringent filtering (Fig.5A), probably attributed to the fact that all sequences are trimmed to 400 bp, so less extensive terminal gaps are created. Clearly, terminal gaps created in alignments of length variable markers represent a theoretical problem in calculating sequence divergence as terminal gaps should represent missing information and not evolutionary differences. This problem is amplified with the use of *each gap*. The *one gap* definition reduces the impact of terminal gaps as every nucleotide is not counted as a difference. Although UPARSE uses the *each gap* definition, this clustering algorithm still had high precision and did not overestimate OTU number as much as the other clustering algorithms did using *each gap*.

Another problem related to identity definitions is that of oversplitting versus undersplitting. OTUs may be oversplit with *each gap* because ribosomal markers are present in multiple gene copies (Bik et al. 2012) and intragenomic length variation between gene copies is common (McTaggart and Crease 2005; James et al. 2009). For example, under relaxed filtering and clustering with mothur, six OTUs were produced for *Centropages abdominalis* with *each gap*, where a single OTU was produced for this species with *no gaps* and *one gap*. Using *each gap* contributes to oversplitting of OTUs and is probably less appropriate because a multinucleotide indel likely represents a single evolutionary event. Conversely, not including gaps as evolutionary differences as with *no gaps* could reduce taxonomic resolution making it difficult to distinguish closely related species. However, in our study with the taxa we used, the exclusion of gaps did not cause any loss of taxonomic resolution (undersplitting did not occur with *no gaps*). The *one gap* definition is a seemingly suitable compromise between *each gap* and *no gaps,* as it retains the useful information of the presence of gaps (retaining taxonomic resolution) but reduces oversplitting by treating gaps as single evolutionary events.

The sequence identity definition for pairwise comparisons is used to assign sequences to clusters and should reflect real differences between species. However, this parameter is rarely explored by metabarcoding researchers. A theoretical basis for treating gaps in ribosomal markers as single or multiple evolutionary events is lacking (Schloss 2010), but it is highly needed in metabarcoding studies. Different clustering algorithms have different default parameters for identity definitions (Table1), which can greatly impact the outcome of OTU generation as shown in this study. Our results indicate that the *each gap* definition should not be used with ribosomal markers when sequences are not all trimmed to the same length and terminal gaps count because this can highly inflated OTU estimates regardless of the sequencing platform employed.

### Concordance of OTUs and species

OTU number alone is not a satisfactory measure of the ability of different workflows to recover species from the mock community. Assigning OTUs to a taxonomic identification as we did is an effective method to detect species actually present and to examine species richness, facilitating a better comparison between workflows. By identifying OTUs, we were able to distinguish those that did not correspond to a species included in the mock community or those that represented variants of the same species. This is reflected in the precision of the various workflows (number of mock community species actually recovered divided by the total number of OTUs produced), which varied between <0.01 and 0.67. The OTUs that did not correspond to species in the mock community were due to either contamination (including gut contents and parasites) or sequencing artifacts. For example, a Platyhelminthes species was consistently recovered by all workflows even though it was not intentionally included in the mock community, and an algal species was recovered when singletons were retained. Also, multiple OTUs often matched the same species, which could be due to polymorphism between gene copies within an individual. For example the *Corbicula fluminea* individual consistently produced multiple OTUs across different workflows. Ideally, clustering would group these sequence variants into a single OTU, but sometimes this does not occur due to extensive variation. Some taxonomic groups were consistently overestimated based on OTU numbers (e.g., cladocerans and molluscs), while others were consistently underestimated (e.g., harpacticoid copepods). Some species were not recovered because they failed to amplify. Clustering at a lower divergence threshold (1%) allowed for the recovery of three more species that were placed in a cluster with a closely related species when the divergence threshold was set to 3% (Table S3), indicating undersplitting with 3%. This reflects a limitation of metabarcoding and the OTU approach in distinguishing some closely related species. Even with mock communities, there is the possibility of bias in DNA extraction and PCR amplification, which can cause missing sequence information for some species or skew the relative read abundance of others. Despite these caveats, mock communities provide insight into how to evaluate natural samples.

### Natural community

The OTU numbers produced from the natural zooplankton community largely mirrored the patterns of the OTU numbers recovered from the mock community for the different workflows (Table4). The most stringent workflow recovered 22 OTUs, while the most relaxed workflow recovered 22,191 OTUs. In this case including singletons caused an increase in OTU number by up to two orders of magnitude compared to the same workflow with singletons removed. This is an even greater difference than what we found for the mock community, probably due to variable species abundances and community complexity, in addition to potential differences in sequence quality and read abundance compared to the mock community (Table4). Corresponding with results from the mock community, mothur and UCLUST produced similar OTU numbers to one another, and UPARSE produced lower numbers in all cases. OTU numbers using each of the identity definitions also showed similar patterns to the results for the mock community (Table4). As with the mock community, there is always a chance that not all species present in the sample will be successfully amplified and recovered. Results from stringent procedures likely reflect a more accurate representation of the diversity of the sample, while possibly oversplitting some species or missing a few rare biological sequences. This suggests that applying the most accurate workflow we found from the mock community on natural zooplankton communities (for which the ground truth species composition is not known) should produce accurate biodiversity estimates.

## Conclusions

Metabarcoding results based on length variable regions, such as 18S, are strongly influenced by the data processing workflow used. Our results indicate that the choice of data filtering, clustering algorithms, and specific parameters has significant impact on the biodiversity estimates generated with metabarcoding data. Overall, we found a very large variation in the number of OTUs produced, ranging from 60 to 5068 for the mock community, with some workflows greatly oversplitting OTUs. This variation was largely produced by the interaction of different filtering regimes – particularly trimming sequences to a uniform length or retaining sequences of variable length – with the method of treating gaps in the alignment when calculating sequence divergence. Treating each nucleotide in a gap as a difference (*each gap*) resulted in great overestimation of OTU number, and this was largely due to terminal gaps created in alignments, which are treated as differences in the calculation of sequence divergence and not as missing data with this definition. Relaxed procedures including filtering and the inclusion of singletons allowed for the detection of only a few species not identified with stringent procedures (e.g., 42 vs. 40 species), but overestimated diversity in terms of OTU number, sometimes extensively (e.g., 114 vs. 68 OTUs). The clustering algorithm UPARSE was more precise and produced more consistent OTU numbers even with relaxed filtering and when including singletons, whereas mothur and UCLUST produced varied and inflated OTU numbers. UPARSE produced OTU numbers in closest concordance with the number of species in the mock community than the other clustering algorithms, even though it uses *each gap*. When methods were tested on a natural community, OTU numbers showed patterns similar to our mock community results, supporting that our findings are applicable to natural communities typically sampled for applications such as biodiversity assays. We suggest that analysis methods should be considered carefully and be tailored to the purpose of the study. If the research goal is to accurately describe biological diversity using OTUs and avoid gross overestimation of species numbers, a stringent approach is more appropriate. However, if the research goal is to identify low abundance species such as those that may be endangered or new invaders, a more relaxed approach could be more sensitive, providing the researcher is prepared to reconcile the effects of artifacts. The metabarcoding field benefits from awareness of the impacts of data processing procedures on biodiversity estimates, including specific parameters. For markers that contain extensive length variation, the proper treatment of gaps and the awareness of terminal gaps are essential to ensure that the clustering algorithm implemented is not generating gross overestimates of biodiversity.

## Data Accessibility

Mock community and natural community sequence data were deposited in the Sequence Read Archive of NCBI (http://www.ncbi.nlm.nih.gov/sra) under the accession numbers specified in the text.

## Supporting Information

**Table S1.** Species included in the mock community.

**Table S2.** The reference database used for BLAST.

**Table S3.** Species that were recovered when clustering at 1% divergence with an explanation of what species they clustered with at 3% divergence.

## References

[b1] Altschul SF, Gish W, Miller W, Myers EW, Lipman DJ (1990). Basic local alignment search tool. J. Mol. Biol.

[b2] Bachy C, Dolan JR, Lopez-Garcia P, Deschamps P, Moreira D (2013). Accuracy of protist diversity assessments: morphology compared with cloning and direct pyrosequencing of 18S rRNA genes and ITS regions using the conspicuous tintinnid ciliates as a case study. ISME J.

[b3] Barriuso J, Valverde JR, Mellado RP (2011). Estimation of bacterial diversity using next generation sequencing of 16S rDNA: a comparison of different workflows. BMC Bioinformatics.

[b4] Behnke A, Engel M, Christen R, Nebel M, Klein RR, Stoeck T (2011). Depicting more accurate pictures of protistan community complexity using pyrosequencing of hypervariable SSU rRNA gene regions. Environ. Microbiol.

[b5] Bik HM, Porazinska DL, Creer S, Gregory Caporaso J, Knight R, Kelley Thomas W (2012). Sequencing our way towards understanding global eukaryotic biodiversity. Trends Ecol. Evol.

[b6] Bonder MJ, Abeln S, Zaura E, Brandt BW (2012). Comparing clustering and pre-processing in taxonomy analysis. Bioinformatics.

[b7] Cai Y, Sun Y (2011). ESPRIT-Tree: hierarchical clustering analysis of millions of 16S rRNA pyrosequences in quasilinear computational time. Nucleic Acids Res.

[b8] Caporaso JG, Kuczynski J, Stombaugh J, Bittinger K, Bushman FD, Costello EK (2010). QIIME allows analysis of high-throughput community sequencing data. Nat. Methods.

[b9] Chariton AA, Ho KT, Proestou D, Bik H, Simpson SL, Portis LM (2014). A molecular-based approach for examining responses of eukaryotes in microcosms to contaminant-spiked estuarine sediments. Environ. Toxicol. Chem.

[b10] Chen W, Cheng Y, Zhang CK, Zhang S, Zhao H (2013a). MSClust: a multi-seeds based clustering algorithm for microbiome profiling using 16S rRNA sequence. J. Microbiol. Methods.

[b11] Chen W, Zhang CK, Cheng Y, Zhang S, Zhao H (2013b). A comparison of methods for clustering 16S rRNA sequences into OTUs. PLoS ONE.

[b12] Choe CP, Hancock JM, Hwang UW, Kim W (1999). Analysis of the primary sequence and secondary structure of the unusually long SSU rRNA of the soil bug, *Armadillidium vulgare*. J. Mol. Evol.

[b13] Cole JR, Wang Q, Cardenas E, Fish J, Chai B, Farris RJ (2009). The ribosomal database project: improved alignments and new tools for rRNA analysis. Nucleic Acids Res.

[b14] Crease TJ, Taylor DJ (1998). The origin and evolution of variable-region helices in V4 and V7 of the small-subunit ribosomal RNA of branchiopod crustaceans. Mol. Biol. Evol.

[b16] Edgar RC (2010). Search and clustering orders of magnitude faster than BLAST. Bioinformatics.

[b17] Edgar RC (2013). UPARSE: highly accurate OTU sequences from microbial amplicon reads. Nat. Methods.

[b18] Edgar RC, Haas BJ, Clemente JC, Quince C, Knight R (2011). UCHIME improves sensitivity and speed of chimera detection. Bioinformatics.

[b19] Egge E, Bittner L, Andersen T, Audic S, de Vargas C, Edvardsen B (2013). 454 pyrosequencing to describe microbial eukaryotic community composition, diversity and relative abundance: a test for marine haptophytes. PLoS ONE.

[b20] Englisch U, Coleman C, Wagele J (2003). First observations on the phylogeny of the families Gammaridae, Crangonyctidae, Melitidae, Niphargidae, Megaluropidae and Oedicerotidae (Amphipoda, Crustacea), using small subunit rDNA gene sequences. J. Nat. Hist.

[b21] Falgueras J, Lara AJ, Fernandez-Pozo N, Canton FR, Perez-Trabado G, Gonzalo Claros M (2010). SeqTrim: a high-throughput pipeline for pre-processing any type of sequence read. BMC Bioinformatics.

[b22] Fonseca VG, Carvalho GR, Sung W, Johnson HF, Power DM, Neill SP (2010). Second-generation environmental sequencing unmasks marine metazoan biodiversity. Nat. Commun.

[b23] Ghodsi M, Liu B, Pop M (2011). DNACLUST: accurate and efficient clustering of phylogenetic marker genes. BMC Bioinformatics.

[b24] Goeker M, Grimm GW, Auch AF, Aurahs R, Kucera M (2010). A clustering optimization strategy for molecular taxonomy applied to planktonic foraminifera SSU rDNA. Evol. Bioinform.

[b25] Hancock J (1995). The contribution of DNA slippage to eukaryotic nuclear 18s-ribosomal-RNA evolution. J. Mol. Evol.

[b26] Hao X, Jiang R, Chen T (2011). Clustering 16S rRNA for OTU prediction: a method of unsupervised Bayesian clustering. Bioinformatics.

[b27] Hebert PDN, Cywinska A, Ball SL, deWaard JR (2003). Biological identification through DNA barcodes. Proc. R. Soc. Lond. B Biol. Sci.

[b28] Huse SM, Huber JA, Morrison HG, Sogin ML, Mark Welch D (2007). Accuracy and quality of massively parallel DNA pyrosequencing. Genome Biol.

[b29] Huse SM, Welch DM, Morrison HG, Sogin ML (2010). Ironing out the wrinkles in the rare biosphere through improved OTU clustering. Environ. Microbiol.

[b30] Hwang U, Ree H, Kim W (2000). Evolution of hypervariable regions, V4 and V7, of insect 18S rRNA and their phylogenetic implications. Zoolog. Sci.

[b31] James SA, O'Kelly MJ, Carter DM, Davey RP, van Oudenaarden A, Roberts IN (2009). Repetitive sequence variation and dynamics in the ribosomal DNA array of *Saccharomyces cerevisiae* as revealed by whole-genome resequencing. Genome Res.

[b32] Jiang X, Zhang H, Sheng H, Wang Y, He Y, Zou F (2012). Two-stage clustering (TSC): a pipeline for selecting operational taxonomic units for the high-throughput sequencing of PCR amplicons. PLoS ONE.

[b33] Katoh K, Standley DM (2013). MAFFT multiple sequence alignment software version 7: improvements in performance and usability. Mol. Biol. Evol.

[b35] Kunin V, Engelbrektson A, Ochman H, Hugenholtz P (2010). Wrinkles in the rare biosphere: pyrosequencing errors can lead to artificial inflation of diversity estimates. Environ. Microbiol.

[b36] Li W, Godzik A (2006). Cd-hit: a fast program for clustering and comparing large sets of protein or nucleotide sequences. Bioinformatics.

[b37] Mahé F, Rognes T, Quince C, de Vargas C, Dunthorn M (2014). Swarm: robust and fast clustering method for amplicon-based studies. PeerJ.

[b38] Mariette J, Noirit C, Klopp C (2011). Assessment of replicate bias in 454 pyrosequencing and a multi-purpose read-filtering tool. BMC Res. Notes.

[b39] May A, Abeln S, Crielaard W, Heringa J, Brandt BW (2014). Unraveling the outcome of 16S rDNA-based taxonomy analysis through mock data and simulations. Bioinformatics.

[b40] McPherson JD (2009). Next-generation gap. Nat. Methods.

[b41] McTaggart SJ, Crease TJ (2005). Selection of the structural stability of a ribosomal RNA expansion segment in *Daphnia obtusa*. Mol. Biol. Evol.

[b43] Pandey RV, Nolte V, Schlotterer C (2010). CANGS: a user-friendly utility for processing and analyzing 454 GS-FLX data in biodiversity studies. BMC Res. Notes.

[b44] Pawlowski J, Esling P, Lejzerowicz F, Cedhagen T, Wilding TA (2014). Environmental monitoring through protist next-generation sequencing metabarcoding: assessing the impact of fish farming on benthic foraminifera communities. Mol. Ecol. Resour.

[b45] Pompanon F, Deagle BE, Symondson WOC, Brown DS, Jarman SN, Taberlet P (2012). Who is eating what: diet assessment using next generation sequencing. Mol. Ecol.

[b46] Quast C, Pruesse E, Yilmaz P, Gerken J, Schweer T, Yarza P (2013). The SILVA ribosomal RNA gene database project: improved data processing and web-based tools. Nucleic Acids Res.

[b47] Quince C, Lanzen A, Curtis TP, Davenport RJ, Hall N, Head IM (2009). Accurate determination of microbial diversity from 454 pyrosequencing data. Nat. Methods.

[b48] Quince C, Lanzen A, Davenport RJ, Turnbaugh PJ (2011). Removing noise from pyrosequenced amplicons. BMC Bioinformatics.

[b49] Russell DJ, Way SF, Benson AK, Sayood K (2010). A grammar-based distance metric enables fast and accurate clustering of large sets of 16S sequences. BMC Bioinformatics.

[b50] Schloss PD (2010). The effects of alignment quality, distance calculation method, sequence filtering, and region on the analysis of 16S rRNA gene-based studies. PLoS Comput. Biol.

[b51] Schloss PD, Westcott SL, Ryabin T, Hall JR, Hartmann M, Hollister EB (2009). Introducing mothur: open-source, platform-independent, community-supported software for describing and comparing microbial communities. Appl. Environ. Microbiol.

[b52] Sun Y, Cai Y, Liu L, Yu F, Farrell ML, McKendree W (2009). ESPRIT: estimating species richness using large collections of 16S rRNA pyrosequences. Nucleic Acids Res.

[b53] Sun Y, Cai Y, Huse SM, Knight R, Farmerie WG, Wang X (2012). A large-scale benchmark study of existing algorithms for taxonomy-independent microbial community analysis. Brief. Bioinform.

[b54] Tedersoo L, Nilsson RH, Abarenkov K, Jairus T, Sadam A, Saar I (2010). 454 Pyrosequencing and Sanger sequencing of tropical mycorrhizal fungi provide similar results but reveal substantial methodological biases. New Phytol.

[b55] Wang X, Yao J, Sun Y, Mai V (2013). M-pick, a modularity-based method for OTU picking of 16S rRNA sequences. BMC Bioinformatics.

[b56] Wuyts J, De Rijk R, Van de Peer Y, Pison G, Rousseeuw P, De Wachter R (2000). Comparative analysis of more than 3000 sequences reveals the existence of two pseudoknots in area V4 of eukaryotic small subunit ribosomal RNA. Nucleic Acids Res.

[b57] Yang CX, Ji YQ, Wang XY, Yang CY, Yu DW (2013). Testing three pipelines for 18S rDNA-based metabarcoding of soil faunal diversity. Sci. China-Life Sci.

[b58] Zhan A, Hulak M, Sylvester F, Huang X, Adebayo AA, Abbott CL (2013). High sensitivity of 454 pyrosequencing for detection of rare species in aquatic communities. Methods Ecol. Evol.

