# Supplementary material for: Toward accurate molecular identification of species in complex environmental samples: testing the performance of sequence filtering and clustering methods
Source: Ecol Evol. 2015 May 13;5(11):2252–66. doi: 10.1002/ece3.1497 (PMC4461425; doi:10.1002/ece3.1497)
Supplement: Supplementary file 1 [file ece30005-2252-sd1.docx]

**Table S1.** Species included in the mock community. Each species was represented as a single individual with a total of 61 species.

| **Group** | **Species** | **Geographic origin** | **Provider** |
| --- | --- | --- | --- |
| Amphipoda | *Crangonyx spp.* | Unknown | Jonathan Witt |
| Amphipoda | *Gammarus lacustris* | Unknown | Jonathan Witt |
| Amphipoda | *Gammarus lawrencianus* | St. Andrews, Canada | Adriana Radulovici |
| Amphipoda | *Gammarus oceanicus* | Terra Nova, Canada | Adriana Radulovici |
| Amphipoda | *Hyalella azteca* | Unknown | Jennifer Adams |
| Amphipoda | *Hyalella* clade 1 | Unknown | Jonathan Witt |
| Amphipoda | *Hyalella* clade 8 | Unknown | Jonathan Witt |
| Amphipoda | *Hyperia galba* | Resolute, Canada | Adriana Radulovici |
| Amphipoda | *Hyperoche medusarum* | Resolute, Canada | Adriana Radulovici |
| Amphipoda | *Themisto libellula* | Chukchi Sea, USA | Adriana Radulovici |
| Anostraca | *Artemia franscicana* | Unknown | Jennifer Adams |
| Anostraca | *Artemia salina* | Unknown | Live Aquaria |
| Cirripedia | *Balanus crenatus* | Puget Sound, USA | Hilary Hayford |
| Cirripedia | *Balanus glandula* | Puget Sound, USA | Hilary Hayford |
| Cirripedia | *Chthamalus dalli* | Puget Sound, USA | Hilary Hayford |
| Cladocera | *Bosmina longirostris* | Lake Erie, Nanticoke, Canada | Colin Van Overdijk |
| Cladocera | *Bythotrephes longimanus* | Huntsville, Canada | Colin Van Overdijk |
| Cladocera | *Ceriodaphnia lacustris* | Nanticoke, Canada | Colin Van Overdijk |
| Cladocera | *Daphnia obtusa* | Pallanza, Piemonte, Italy | Alessandra Loria |
| Cladocera | *Daphnia parvula* | Huntsville, Canada | Colin Van Overdijk |
| Cladocera | *Daphnia pulex* | Champaign- Urbana, USA | Tiffany Chin |
| Cladocera | *Daphnia pulicaria* | Champaign- Urbana, USA | Tiffany Chin |
| Cladocera | *Diaphanosoma brachyurum* | Nanticoke, Canada | Colin Van Overdijk |
| Cladocera | *Holopedium gibberum* | Huntsville, Canada | Colin Van Overdijk |
| Cladocera | *Leptodora kindti* | Lake Erie, Nanticoke, Canada | Colin Van Overdijk |
| Cladocera | *Polyphemus pediculus* | Lake Huron, Canada | Colin Van Overdijk |
| Copepoda (calanoid) | *Leptodiaptomus minutus* | Lake Huron, Canada | Colin Van Overdijk |
| Copepoda (calanoid) | *Limnocalanus macrurus* | Lake Erie, Nanticoke, Canada | Colin Van Overdijk |
| Copepoda (calanoid) | *Acartia tonsa* | Hawkesbury, Canada | Siobhan Curry |
| Copepoda (calanoid) | *Calanus finmarchicus* | Frobisher Bay Iqaluit, Canada | Rob Young |
| Copepoda (calanoid) | *Centropages abdominalis* | Burrard Inlet Vancouver, Canada | Rob Young |
| Copepoda (calanoid) | *Eurytemora affinis* | Quebec City, Canada | Elizabeta Briski |
| Copepoda (calanoid) | *Microcallanus pusillus* | Strait of Canso Hawkesbury,Canada | Rob Young |
| Copepoda (calanoid) | *Pseudocalanus mimus* | Nanticoke, Canada | Siobhan Curry |
| Copepoda (cyclopoid) | *Acanthocyclops vernalis* | Thunder Bay, Canada | Siobhan Curry |
| Copepoda (cyclopoid) | *Corycaeus anglicus* | Strait of Georgia Robert's Bank, Canada | Rob Young |
| Copepoda (cyclopoid) | *Eucyclops speratus* | Lake Erie, Nanticoke, Ontario | Colin Van Overdijk |
| Copepoda (cyclopoid) | *Macrocyclops albidus* | North Frontenac, Canada | Colin Van Overdijk |
| Copepoda (cyclopoid) | *Oithona atlantica* | Burrard Inlet Vancouver, Canada | Rob Young |
| Copepoda (harpacticoid) | *Clytemnestra scutellata* | Victoria, Canada | Siobhan Curry |
| Copepoda (harpacticoid) | Tachidiidae | Iqualuit, Canada | Siobhan Curry |
| Copepoda (harpacticoid) | *Tisbe furcata* | Hudson Strait Deception Bay, Canada | Rob Young |
| Copepoda (harpacticoid) | *Zaus abbreviatus* | Hudson Strait Deception Bay, Canada | Rob Young |
| Decapoda | *Carcinus maenas* | Strait of Canso Hawkesbury, Canada | Rob Young |
| Decapoda | Caridea | Deception Bay, Canada | Siobhan Curry |
| Decapoda | Crangonidae | Nanticoke, Canada | Siobhan Curry |
| Decapoda | Grapsidae | Vancouver, Canada | Siobhan Curry |
| Decapoda | Hippolytidae | Nanticoke, Canada | Siobhan Curry |
| Decapoda | Majidae | Frobisher Bay Iqaluit, Canada | Rob Young |
| Decapoda | *Neotrypaea californiensis* | Nanticoke, Canada | Siobhan Curry |
| Decapoda | Xanthidae | Strait of Georgia Nanaimo, Canada | Rob Young |
| Mollusca | *Corbicula fluminea* | St Lawrence River, Quebec, Canada | Emilija Cvetanovska |
| Mollusca | *Dreissena polymorpha* | Quebec City, Canada | Elizabeta Briski |
| Mollusca | *Limacina helicina* | Hudson Strait Deception Bay, Canada | Rob Young |
| Mollusca | *Limnoperna fortunei* | Argentina | Sara Ghabooli |
| Mollusca | *Mytilus edulis* | Halifax, Canada | Farrah Chan |
| Mollusca | *Nassarius distortus* | Unknown | Live Aquaria |
| Mollusca | *Nerita spp.* | Unknown | Live Aquaria |
| Mollusca | Pteropoda | Unknown | Siobhan Curry |
| Tunicate | *Ciona intestinalis* | Cardigan River, Canada | Sara Ghabooli |
| Tunicate | *Oikopleura labradoriensis* | Foxe Basin Steensby Inlet, Canada | Siobhan Curry |
