# Supplementary material for: Toward accurate molecular identification of species in complex environmental samples: testing the performance of sequence filtering and clustering methods
Source: Ecol Evol. 2015 May 13;5(11):2252–66. doi: 10.1002/ece3.1497 (PMC4461425; doi:10.1002/ece3.1497)
Supplement: Supplementary file 2 [file ece30005-2252-sd2.docx]

**Table S2**. The reference database used for BLAST. Only species that amplified from the community are shown.

| **Group** | **Identification** | **Representative sequence in database** | **Percent ID with representative sequence** |
| --- | --- | --- | --- |
| Amphipoda | *Crangonyx spp.* | *Crangonyx forbesi* | 92% |
| Amphipoda | *Gammarus lawrencianus* | *Gammarus bousfieldi* | 100% |
| Amphipoda | *Gammarus oceanicus* | *Gammarus setosus* | 100% |
| Amphipoda | *Hyalella azteca* | *Hyalella azteca* | 99.8% |
| Amphipoda | *Hyalella sp* clade 1 / *Hyalella* clade 8 | *Hyalella sp.* | 99.79% |
| Amphipoda | *Hyperia galba* | *Hyperia galba* | 100% |
| Amphipoda | *Hyperoche medusarum* | *Hyperoche capucinus* | 100% |
| Amphipoda | *Themisto libellula* | *Themisto libellula* | 98.67% |
| Anostraca | *Artemia salina* | *Artemia salina* | 100% |
| Cirripedia | *Balanus crenatus* | *Balanus crenatus* | 99.13% |
| Cirripedia | *Balanus glandula* | *Balanus glandula* | 100% |
| Cirripedia | *Chthamalus dalli* | *Chthamalus fragilis* | 98.26% |
| Cladocera | *Bosmina longirostris* | *Bosmina longirostris* | 100% |
| Cladocera | *Bythotrephes longimanus* | *Bythotrephes cederstroemi* | 100% |
| Cladocera | *Daphnia obtusa / Daphnia parvula* | *Dapnhia longiremis* | 100% |
| Cladocera | *Daphnia pulex* | *Daphnia pulex* | 100% |
| Cladocera | *Diaphanosoma brachyurum* | *Diaphanosoma sp* | 100% |
| Cladocera | *Holopedium gibberum* | *Holopedium gibberum* | 100% |
| Cladocera | *Leptodora kindti* | *Leptodora kindtii* | 98.51% |
| Cladocera | *Polyphemus pediculus* | *Polyphemus pediculus* | 100% |
| Copepoda (calanoid) | *Leptodiaptomus minutus* | *Leptodiaptomus ashlandi* | 100% |
| Copepoda (calanoid) | *Acartia tonsa* | *Acartia tonsa* | 100% |
| Copepoda (calanoid) | *Calanus finmarchicus* | *Calanus pacificus* | 99.03% |
| Copepoda (calanoid) | *Centropages abdominalis* | *Centropages hamatus* | 100% |
| Copepoda (calanoid) | *Eurytemora affinis* | *Eurytemora affinis* | 100% |
| Copepoda (calanoid) | *Pseudocalanus mimus* | *Pseudocalanua elongatus* | 100% |
| Copepoda (cyclopoid) | *Corycaeus anglicus* | *Corycaeus speciosus* | 96.12% |
| Copepoda (cyclopoid) | *Eucyclops speratus* | *Euclyclops macruroides* | 100% |
| Copepoda (cyclopoid) | *Macrocyclops albidus* | *Macrocyclops albidus* | 100% |
| Copepoda (harpacticoid) | *Clytemnestra scutellata* | *Alteuthellopsis sp* | 91.43% |
| Copepoda (harpacticoid) | *Tisbe furcata* | *Tisbe tenera* | 100% |
| Copepoda (harpacticoid) | *Zaus abbreviatus* | *Zaus caeruleus* | 99.68% |
| Decapoda | Caridea / Hippolytidae | *Thoralus cranchii* | 97.73% |
| Decapoda | Crangonidae | *Neocrangon sagamiensis* | 99.12% |
| Decapoda | Grapsidae | *Cyclograpsus cinereus* | 99.38% |
| Decapoda | *Neotrypaea californiensis* | *Neotrypaea californiensis* | 98.17% |
| Mollusca | *Corbicula fluminea* | *Corbicula fluminea* | 99.68% |
| Mollusca | *Limacina helicina /* Pteropoda | *Stylocheilus longicauda* | 98.11% |
| Mollusca | *Mytilus edulis* | *Mytilus trossulus* | 100% |
| Mollusca | *Nassarius distortus* | *Nassarius festivus* | 100% |
| Mollusca | *Nerita spp.* | *Bathynerita naticoida* | 100% |
| Tunicate | *Ciona intestinalis* | *Ciona intestinalis* | 99.35% |
| Tunicate | *Oikopleura labradoriensis* | *Oikopleura labradoriensis* | 94.59% |
