# Supplementary material for: Toward accurate molecular identification of species in complex environmental samples: testing the performance of sequence filtering and clustering methods
Source: Ecol Evol. 2015 May 13;5(11):2252–66. doi: 10.1002/ece3.1497 (PMC4461425; doi:10.1002/ece3.1497)
Supplement: Supplementary file 3 [file ece30005-2252-sd3.docx]

**Table S3**. Species that were recovered when clustering at 1% divergence with an explanation of what species they clustered with at 3% divergence. Reads were filtered with stringent procedures, singletons were included, and clustering was performed with UCLUST.

| Novel species recovered | Similarity to reference database (BLAST) | Species clustered with at 3% divergence | Similarity to seed of this cluster |
| --- | --- | --- | --- |
| *Artemia franciscana* | 99.5% | *Artemia salina* | 98% |
| *Limnocalanus macrurus* | 98% | *Eurytemora affinis* | 98% |
| *Limnoperna fortunei* | 94% | *Mytilus edulis (trossulus)* | 99% |
